# Comparison of post-operative pain between rotary and reciprocating file systems in root canal therapy: An *in vivo* study

**DOI:** 10.6026/973206300222970

**Published:** 2026-05-31

**Authors:** Prasad Chandrakant Ingale, Sulaima Mehar, Proxima Bora, Mohd Zeeshan Ahmad, Jaini Thakkar, Shreya Gill, Pratik Surana

**Affiliations:** 1 Department of Conservative and Endodontics, Bharati Vidyapeeth (Deemed to be University) Dental College and Hospital, Sangli, Maharashtra, India; 2 Department of Conservative Dentistry and Endodontics, Indira Gandhi Institute of Medical Sciences, Patna, Bihar, India; 3 Department of Dentistry, Tezpur Medical College and Hospital, Tezpur, Assam, India; 4 Department of Conservative Dentistry & Endodontics, Rama Dental College Hospital & Research Centre, Kanpur, Uttar Pradesh, India; 5 Department of Conservative Dentistry and Endodontics, Faculty of Dental Science, Dharmsinh Desai University, Nadiad, Gujarat, India; 6 Department of Conservative Dentistry and Endodontics, Winchester Dental Care, Virginia, USA; 7 Department of Pedodontics and Preventive Dentistry, Maitri College of Dentistry and Research Centre, Durg, Chhattisgarh, India

**Keywords:** Pain, post-operative, endodontic files

## Abstract

Post-operative pain following root canal therapy remains a common clinical concern and is frequently associated with apical extrusion of debris, microorganisms and irrigating solutions during canal instrumentation. Therefore, it is of interest to evaluate post-operative pain following instrumentation with Hand ProTaper (control), ProTaper Next (rotary), and Neo-Endo (reciprocating) systems in 45 patients undergoing endodontic treatment. Pain intensity was assessed using the Visual Analogue Scale (VAS) at 6, 24, 48, and 72 hours postoperatively. The reciprocating system (Neo-Endo) demonstrated lower post-operative pain scores compared to the rotary and hand instrumentation groups. Thus, we show that reciprocating instrumentation may be more effective in minimizing post-operative discomfort following root canal therapy.

## Background:

Post-endodontic pain is frequently observed following root canal treatment, with an incidence of 25%-40%, affecting patients regardless of pulpal or periradicular status [1, 2]. The occurrence and intensity of pain are influenced by multiple factors, including mechanical, chemical and microbial irritation during root canal treatment [3, 4]. During chemomechanical preparation the unintended passage of debris, microbes and irrigants into the periapical area can initiate an inflammatory response [5]. This inflammatory process leads to the release of mediators such as prostaglandins, which are primarily responsible for Post-operative pain [6]. In addition, several clinical variables-including gender, tooth type, pre-operative pain, periapical pathology, procedural errors, missed canals, occlusal discrepancies, intracanal medicament extrusion and apical patency have been implicated in contributing to post-treatment discomfort [7, 8]. Recent advancements in endodontic instrumentation have focused on improving cleaning efficiency while minimizing procedural errors and Post-operative complications [9]. Nickel-titanium (NiTi) file systems with different kinematics, namely continuous rotary and reciprocating motion, are widely used in contemporary practice [10]. Reciprocating systems, such as Neo-Endo, operate using alternating rotational movements designed to reduce torsional stress and the risk of instrument fracture [11, 12]. In contrast, rotary systems like ProTaper Next feature a unique off-centered rectangular cross-section that enhances cutting efficiency and facilitates debris removal [13, 14]. The design and motion of endodontic instruments play a crucial role in the extent of apical debris extrusion, which may directly influence Post-operative pain [15]. Although newer systems claim reduced Post-operative discomfort, evidence remains inconclusive and existing pain-control approaches may have limitations and adverse effects [16]. Evaluating the impact of different file systems and their kinematics on Post-operative pain is essential for optimizing patient comfort and treatment success. Therefore, it is of interest to comparatively evaluate post-operative pain following endodontic therapy using ProTaper Next (continuous rotary motion) and Neo-Endo (reciprocating motion) file systems.

## Materials and Methods:

This *in vivo* randomized clinical research was undertaken to evaluate Post-operative pain following root canal therapy using different endodontic file systems. A total of 45 subjects requiring endodontic treatment were selected after obtaining informed consent. Patients aged between 18 and 40 years diagnosed with symptomatic irreversible pulpitis in single-rooted teeth were included in the study. Patients with systemic conditions, pregnancy, periapical abscess, retreatment cases, recent use of analgesics (within 12 hours), or teeth with complex root canal anatomy were excluded. The selected participants were randomly allotted into three equal groups. The Group I was instrumented using hand ProTaper files, while Group II underwent canal preparation with ProTaper Next using continuous rotary motion. Group III was treated using the Neo-Endo file system operating in reciprocating motion. All intervention was carried out by a single skilled clinician. Local anesthesia (2% lidocaine with 1:80,000 epinephrines) was administered and pre-operative occlusal reduction was done to reduce Post-operative discomfort. Access cavity preparation was done using a sterile #2 round bur. Working length was ascertained using an electronic apex locator and verified radiographically. A glide path was established prior to canal preparation and coronal enlargement was performed. Biomechanical preparation was done according to the assigned group using either continuous rotary or reciprocating motion. Irrigation was done with 5 ml of 3% sodium hypochlorite using a 30-gauge side-vented needle during instrumentation, alternating with normal saline, followed by 5 ml of 17% EDTA and a final rinse with 10 ml of saline. All cases were managed in two visits. No intracanal medicament was used between schedules to prevent any effect on Post-operative pain. After cleaning and shaping, access cavities were provisionally sealed with an interim material. At the subsequent visit, canals were obturated using gutta-percha with an appropriate sealer following standard techniques. Post-operative pain was recorded using the Visual Analog Scale at 6, 24, 48 and 72 hours. Patients were instructed to record their pain levels accordingly. A rescue analgesic (ibuprofen 400 mg) was prescribed only if required and its intake was documented. The collected data were statistically analyzed using SPSS software (version 25) intergroup comparisons were performed using appropriate statistical tests such as ANOVA and post-hoc analysis.

## Results:

A total of 45 patients were included in the study and completed all follow-up intervals. Post-operative pain was assessed at 6, 24, 48 and 72 hours using the Visual Analog Scale. The mean pain scores for all groups at different time intervals are presented in. At 6 hours, the highest pain scores were observed in all groups, with the control group (hand ProTaper) showing the greatest pain intensity, followed by the ProTaper Next group, while the Neo-Endo (reciprocating) group demonstrated the lowest pain levels (Table 1). A gradual reduction in pain scores was observed at 24, 48 and 72 hours across all groups. Intergroup comparison showed differences in Post-operative pain among the groups at all-time intervals. Further analysis using Tukey's post-hoc test demonstrated that the Neo-Endo group had significantly lower pain scores compared to both the ProTaper Next and control groups at all evaluated time intervals (Table 2). Overall, the reciprocating system (Neo-Endo) consistently exhibited the lowest Post-operative pain scores, whereas the control group showed the highest pain levels throughout the observation period.

## Discussion:

Post-operative pain following endodontic therapy remains an important concern for both clinicians and patients, as it directly affects patient comfort and perception of treatment success [17]. The present in vivo study evaluated the influence of different instrumentation kinematics continuous rotary and reciprocating motion on Post-operative pain. The findings demonstrated that the reciprocating file system (Neo-Endo) was associated with significantly lower Post-operative pain compared to the continuous rotary system (ProTaper Next) and hand instrumentation. The primary cause of Post-operative pain is believed to be the extrusion of debris, microorganisms and irrigants into the periapical tissues during canal preparation [18]. This extrusion can trigger an acute inflammatory response, leading to the release of mediators such as prostaglandins, which are responsible for pain perception [19]. The extent of debris extrusion is influenced by multiple factors, including instrumentation technique, file design and kinematics [20]. In the current research, the uppermost pain levels were observed at 6 hours Post-operatively across all groups, followed by a gradual reduction at 24, 48 and 72 hours. This trend is consistent with previous studies, which report that Post-operative pain peaks within the first 24 hours and subsequently declines as inflammation subsides and healing progresses. The control group (hand ProTaper) consistently showed higher pain scores, which may be attributed to less efficient debris removal and increased apical extrusion during manual instrumentation. The superior performance of the reciprocating system (Neo-Endo) observed in this study can be explained by its unique kinematics. Reciprocating motion involves alternating clockwise and counterclockwise movements, which reduce the tendency of the instrument to push debris apically. Additionally, the design minimizes torsional stress and promotes coronal transportation of debris, thereby enhancing cleaning efficiency. These factors collectively contribute to reduced periapical irritation and lower Post-operative pain. Supporting this, Eliasz et al. [21] reported that WaveOne extruded less debris compared to ProTaper Next and Twisted Files, while Ehsani et al. also clained that reciprocating systems outperform ProTaper Universal and Mtwo systems in minimizing apical debris extrusion [22]. In contrast, continuous rotary systems like ProTaper Next operate in a single-direction rotational motion, which may act like a screw conveyor, potentially pushing debris toward the apex. Although ProTaper Next has an off-centered rectangular cross-section that improves cutting efficiency and debris clearance, it may still result in comparatively higher apical extrusion than reciprocating systems, thereby contributing to increased Post-operative discomfort. Several studies in the literature have reported similar findings, indicating that reciprocating systems tend to produce less Post-operative pain compared to rotary systems [23]. However, some studies have shown conflicting results, possibly due to variations in study design, sample size, operator skill, irrigation protocols and patient-related factors. The standardized methodology used in the present study, including single-operator treatment, uniform irrigation protocol and controlled variables, strengthens the validity of the findings. Various strategies have been proposed to reduce Post-operative pain, including pre-operative analgesics, use of long-acting anesthetics, corticosteroids, occlusal reduction and modification of instrumentation techniques [24, 25]. In this study, occlusal reduction and standardized irrigation protocols were employed to minimize confounding factors. Additionally, no intracanal medicament was used between visits to eliminate its influence on pain perception [16, 17]. Finally, it can be concluded that Post-operative pain following endodontic treatment may be influenced by the type of endodontic file system used during canal preparation [26, 27]. Differences in instrumentation kinematics and debris extrusion can affect patient discomfort; therefore, selecting an appropriate file system is important for achieving effective canal preparation while minimizing Post-operative pain and improving overall treatment outcomes [28, 29]. In contrast to our study, Poorani *et al.* and da Silveira *et al.* reported comparable postoperative pain outcomes between rotary and reciprocating instrumentation systems, suggesting that the type of kinematics may not significantly influence postoperative pain perception following endodontic treatment [30, 31]. Although the study yielded significant findings, certain limitations should be acknowledged. The sample size was relatively small and only single-rooted teeth were included, which may limit the generalizability of the results. Pain assessment using the Visual Analog Scale is subjective and may vary among individuals. Future studies with larger sample sizes, inclusion of multi-rooted teeth and long-term follow-up are recommended to further confirm these results. Within the constraints of the present study, the reciprocating file system is associated with reduced Post-operative pain compared to continuous rotary instrumentation. The choice of instrumentation technique plays a crucial role in influencing patient comfort and should be considered an important factor in endodontic treatment planning.

## Conclusion:

We show that instrumentation technique plays a crucial role in Post-operative pain following root canal therapy. The reciprocating file system was associated with reduced pain levels compared to both rotary and manual instrumentation. Incorporating reciprocating kinematics in clinical practice may enhance patient comfort and improve overall treatment experience.

## Advancement to knowledge:

This study advances current knowledge by providing clinical evidence on how different endodontic instrumentation systems-rotary and reciprocating-affect. Post-operative pain following root canal therapy, thereby helping clinicians selects file systems that improve patient comfort and treatment outcomes. Furthermore, the findings contribute to evidence-based endodontic practice by highlighting the role of instrumentation kinematics in minimizing Post-operative discomfort and enhancing patient-centered care.

## Figures and Tables

**Table 1:** Comparison of mean Post-operative pain scores at different intervals

**Time**	**Group I**	**Group II**	**Group III**	**p-value**
6 hours	6.20 ± 1.10	5.10 ± 1.00	3.80 ± 0.90	<0.05*
24 hours	5.00 ± 1.00	4.00 ± 0.90	2.90 ± 0.80	<0.05*
48 hours	3.80 ± 0.90	2.90 ± 0.80	1.90 ± 0.70	<0.05*
72 hours	2.60 ± 0.80	1.90 ± 0.70	1.10 ± 0.50	<0.05*
*Significant

**Table 2:** Intergroup comparison of Post-operative pain (Tukey Post-Hoc Test)

**Time Interval**	**Group Comparison**	**Mean Difference**	**p-value**
6 hours	I vs II	1.1	0.021*
	I vs III	2.4	<0.001*
	II vs III	1.3	0.010*
24 hours	I vs II	1	0.030*
	I vs III	2.1	<0.001*
	II vs III	1.1	0.015*
48 hours	I vs II	0.9	0.041*
	I vs III	1.9	<0.001*
	II vs III	1	0.020*
72 hours	I vs II	0.7	0.048*
	I vs III	1.5	<0.001*
	II vs III	0.8	0.030*
*=Significant
